# Bilateral Blistering Rash Following Gastric Bypass Procedure and Mushroom Soup Diet: Hepatitis C Virus-Seronegative Necrolytic Acral Erythema

**DOI:** 10.7759/cureus.63755

**Published:** 2024-07-03

**Authors:** Logan R Smith, Sarah Moore, Brandon Cardon, Sudeep Gaudi, Brooke T Baldwin

**Affiliations:** 1 Department of Dermatology and Cutaneous Surgery, University of South Florida Morsani College of Medicine, Tampa, USA; 2 Department of Pathology and Laboratory Medicine, James A. Haley Veterans' Hospital, Tampa, USA; 3 Department of Dermatology, James A. Haley Veterans’ Hospital, Tampa, USA

**Keywords:** dermatology case report, zinc, necrolytic erythema, nutrition and dermatology, complications gastric bypass surgery, necrolytic acral erythema, derm path

## Abstract

Necrolytic acral erythema (NAE) is an uncommon cutaneous disorder characterized by a symmetric acral distribution of erythematous plaques with underlying epidermal necrosis. While typically presenting in the context of hepatitis C virus (HCV) infection, NAE can also present secondary to nutritional deficiency or systemic disease. We present a case of NAE in a 66-year-old patient with no history of HCV infection status post gastric bypass who had a three-month history of eating only mushroom soup. The patient underwent a punch biopsy and was tested for a variety of nutritional deficiencies. Biopsy demonstrated partial necrosis of the upper epidermis, with subjacent re-epithelialization, squamatization, and vacuolopathy of the basal epidermis. He was treated with zinc replacement therapy after initial trials of tacrolimus and clobetasol were unsuccessful. At follow-up, he had significant improvement of the lesions. This case provides an example of an atypical presentation of NAE in the absence of HCV infection that presented as a complication of gastric bypass-associated nutritional deficiency.

## Introduction

Necrolytic acral erythema (NAE) is part of a family of skin disorders known as necrolytic erythemas that have overlapping clinical findings that are largely categorized based on clinical manifestations, common associations, location of cutaneous eruption, and pattern of lesion evolution. NAE is typically associated with hepatitis C virus (HCV) infection but may also be associated with other systemic diseases such as diabetes mellitus and cirrhosis [1]. The pathogenesis of NAE is relatively nuanced and still unknown. Several researchers have hypothesized different causes of NAE, including hyperglucagonemia, much like the similar entity necrolytic migratory erythema (NME) [2]. It is also occasionally seen in the absence of any known systemic disease or cases presenting with hypoalbuminemia or zinc deficiency [1-3]. NAE has a characteristic distribution of the dorsum of the hands and feet, although involvement of the lower legs has also been described [4]. NAE can be distinguished from NME based on the clinical distribution of skin lesions and NAE’s association with HCV; however, HCV does not need to be present to make a diagnosis of NAE [5,6]. Zinc replacement therapy is the mainstay of treatment, regardless of whether the patient has zinc deficiency [1].

## Case presentation

**Figure 1:**
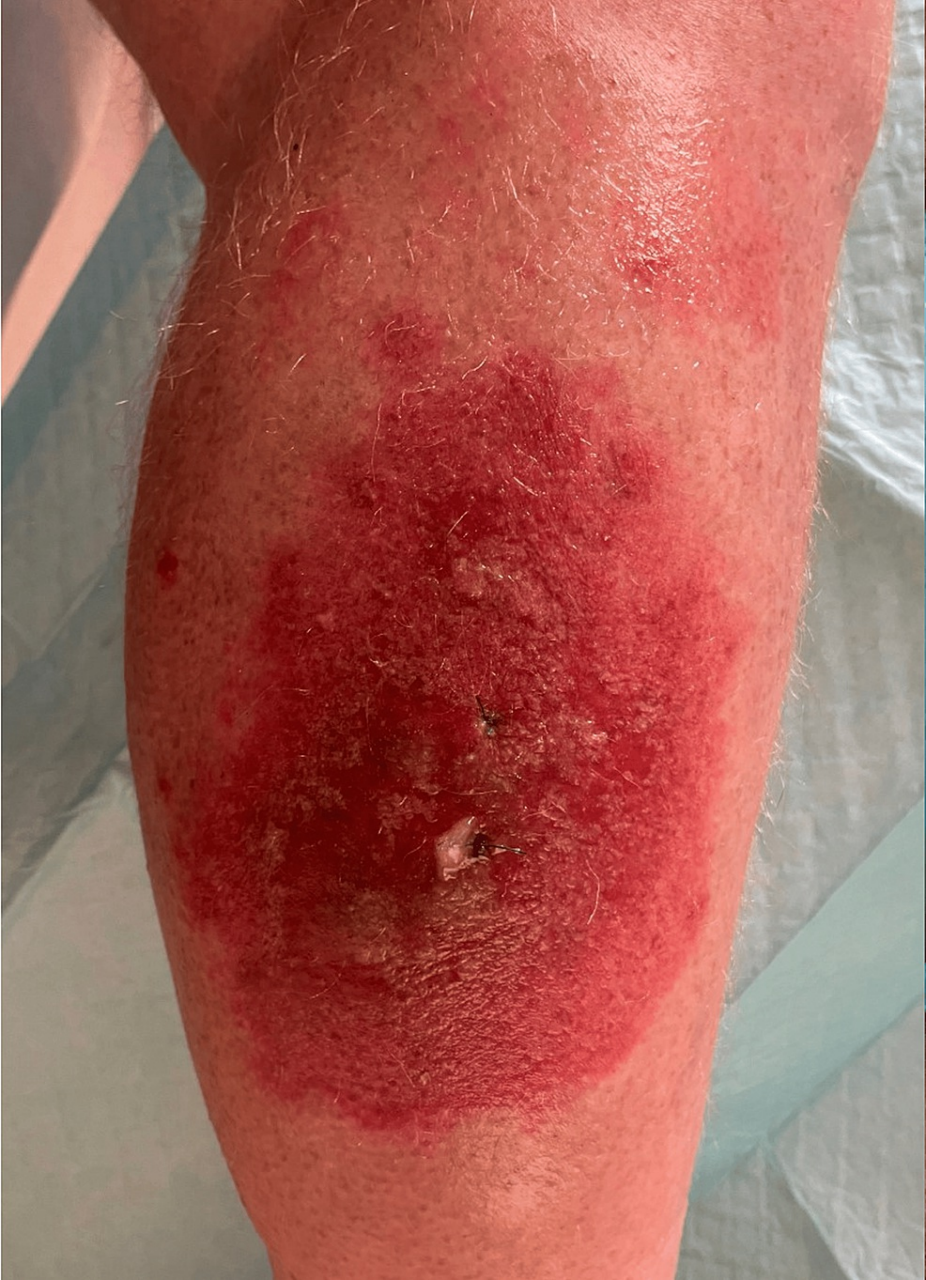
Clinical image of erythematous scaling patches with bulla and tense vesicles on lower legs.

A 66-year-old male presented to our outpatient dermatology clinic with a one-month history of blistering, scaling, and erythematous rash on dorsal hands and lower legs bilaterally (Figure 1). The patient had a gastric bypass surgery six months ago and as a result, can only consume three ounces of food at a time. Contrary to the advice of his postoperation bariatric nutritionist, the patient was following a self-prescribed diet of only mushroom soup. He was taking a multivitamin but discontinued use after the appearance of his rash. Labs showed vitamin A deficiency, hypoalbuminemia, mild zinc deficiency, and hyperbilirubinemia. The lesions were initially treated with topical tacrolimus and clobetasol propionate, but these interventions were unsuccessful. On follow-up, after a seven-day course of oral prednisone, the patient’s rash was worsening and spreading.

**Figure 2:**
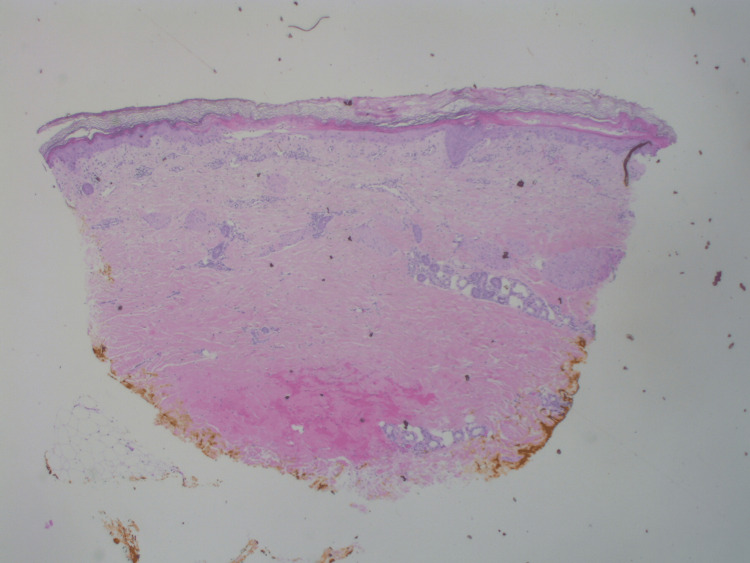
Low-power view (hematoxylin & eosin, original magnification x20) of the punch biopsy specimen.

**Figure 3:**
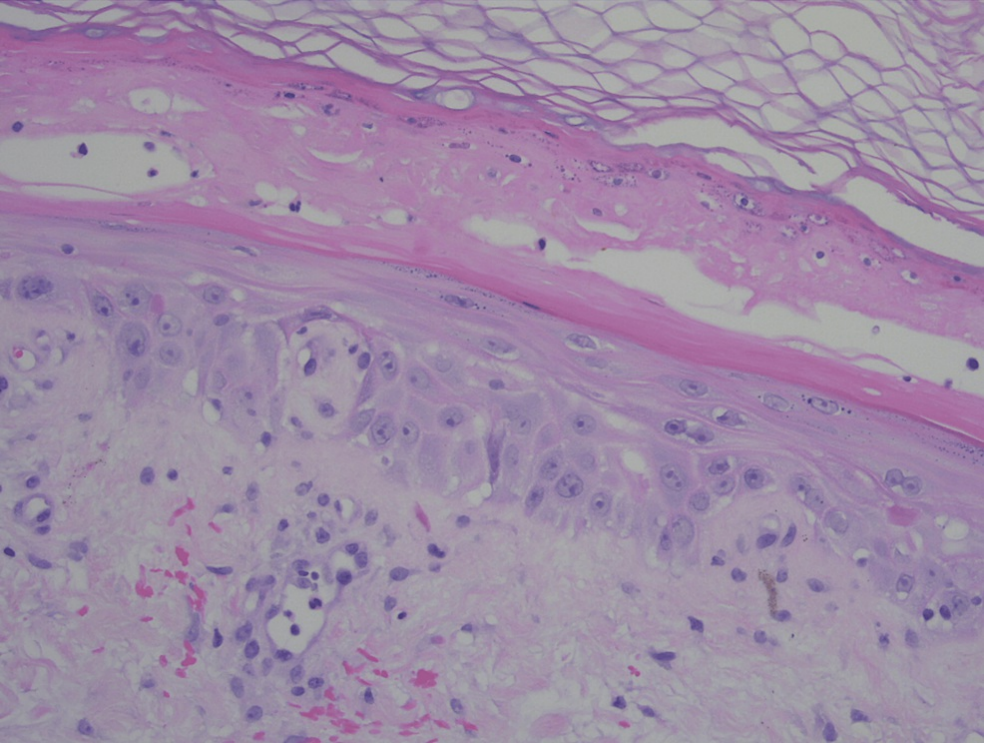
High-power view (hematoxylin & eosin, original magnification x200) reveals partial necrosis with intermixed neutrophils of the upper epidermis. Subjacent re-epithelialization is present, with squamatization and vacuolopathy of the basal epidermis.

A physical exam showed erythematous patches with bulla and tense vesicles bilaterally on the dorsum of the hands and bilateral lower legs. Punch biopsy was performed for histopathological study. Histologic sections show hyperkeratosis, pallor of the upper epidermis, interface changes, and a superficial and mid-perivascular inflammatory infiltrate (Figure 2). Higher-power examination showed partial necrosis of the upper epidermis and squamatization and vacuolopathy of the basal epidermis (Figure 3).

These findings, coupled with the patient’s previous medical history, were consistent with NAE. The patient had a negative HCV result two years prior to the rash onset and at the time of rash presentation, repeat HCV labs were nonreactive. Given that our patient had no history of HCV infection or other liver pathology, the presence of NAE is most likely related to nutritional deficiency, secondary to gastric bypass surgery.

The patient’s self-prescribed diet of mushroom soup was likely void of the necessary quantity of zinc and other nutrients. Notably, lab values illustrated low levels of vitamin A (18 mcg/dL), vitamin D (27.1 ng/mL), and prealbumin (16.4 mg/dL), and low-normal levels of zinc (65 mcg/dL; normal: 66-106 mcg/dL). The patient had normal alkaline phosphatase levels (70 mg/dL) and elevated bilirubin (total bilirubin: 1.8 mg/dL; direct: 0.8 mg/dL; indirect: 1.0 mg/dL). The patient was taking a multivitamin supplement that was prescribed by the bariatric nutritionist but discontinued its use upon the onset of cutaneous eruption. The patient’s adherence to this vitamin is unknown. The multivitamin was a standard Veterans Affairs pharmacy-dispensed complete multivitamin supplement for adults.

**Figure 4:**
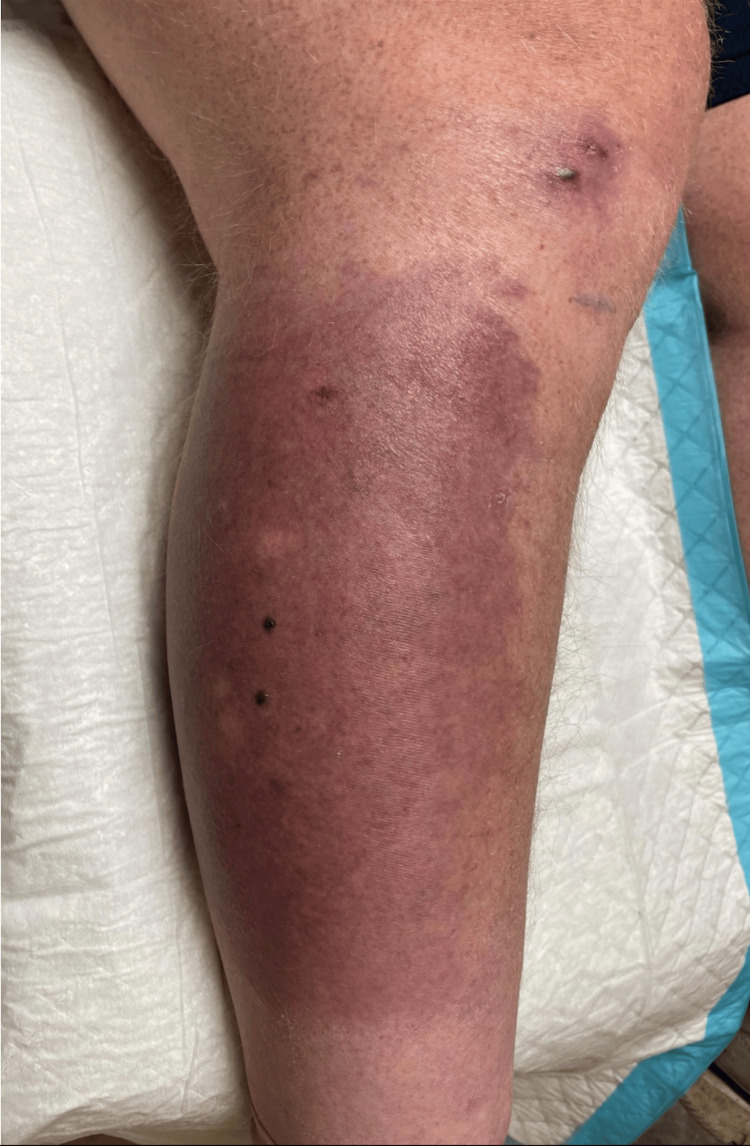
Clinical image of the lesion resolution following three weeks of treatment with zinc supplementation.

Following diagnosis with NAE, the patient was instructed to restart his multivitamin and was prescribed daily zinc supplementation of 400 mg. Although the patient received extensive postoperation bariatric nutrition guidance and was prescribed a high-protein diet, folic acid, iron, and a multivitamin, the patient likely required additional guidance with his diet. Thus, the patient was referred to nutrition for consultation regarding modifying his postoperative diet. After treatment with zinc supplementation, the patient’s lesions resolved completely. Figure 4 shows the lesion resolution following three weeks of treatment with zinc supplementation.

## Discussion

NAE has been known to be a cutaneous indication of HCV infection and the majority of NAE cases present with HCV [1]. However, a few case reports have indicated that NAE can rarely present in the absence of HCV [1,5,7,8]. Clinically, NAE presents as violaceous well-demarcated plaques with possible pustules and vesicles over primarily acral areas (dorsum of hands and feet) with sparing of hair and nails. Some cases, however, have described NAE lesions as reaching the lower legs as well [4,9]. While NAE is typically asymptomatic, occasionally, it can present with an intense burning sensation. Pathogenesis of NAE is currently unknown but has been hypothesized to be related to hyperglucagonemia, hypoalbuminemia, zinc deficiency, and diabetes. Hypoalbuminemia leads to inflammation seen in NAE because albumin can sequester fatty acids, thus preventing fatty acid degradation into prostaglandins; additionally, hypoalbuminemia can lead to zinc deficiency since albumin is a prominent carrier of zinc [2]. Clinical-pathological correlation is essential to arrive at the proper NAE diagnosis. Histopathology for NAE classically shows mid and upper epidermal pallor, which progresses to necrosis and separation. The histopathologic findings in this case also showed interface changes of basilar vacuolopathy and squamatization. While not a typical association, such interface changes have been reported in association with necrolytic erythemas on occasion [10,11]. Whether these interface changes represent a genuine immune-mediated phenomenon or secondary changes of keratinocytic dysmaturation in the setting of nutritional deficiency is debatable.

Other conditions that are included in the differential diagnosis of NAE include zinc-responsive acral hyperkeratosis, acrodermatitis enteropathica (AE), acquired zinc deficiency, primary pellagra, and progressive symmetric erythrokeratoderma. Zinc-responsive acral hyperkeratosis is an entity that has a similar clinical presentation as NAE and can only be differentiated based on histology. Histopathology can demonstrate acanthosis, hyperkeratosis, focal parakeratosis, and perivascular inflammatory infiltrate; however, necrosis and vacuolization are not typically seen in zinc-responsive acral hyperkeratosis [12]. The necrosis and dyskeratotic keratinocytes seen in our patient point toward a diagnosis of HCV-seronegative NAE.

AE is typically an autosomal-recessive genetic disorder of zinc metabolism that presents in infants with acral and facial erythematous plaques, alopecia, and diarrhea. AE stems from a mutation in the zinc transporter gene, SLC39A4 [13]. Similarly to other necrolytic erythemas, AE presents with spongiosis, neutrophilic infiltrate, keratinocyte necrosis, and reticular degeneration.

Our patient’s age at presentation may suggest that the genetic form of AE is an unlikely diagnosis; however, there is an acquired form of AE that appears in the context of hypozincemia [13]. Acquired zinc deficiency, or acquired AE, usually presents with a facial, acral, and anogenital cutaneous distribution of well-demarcated, erythematous, eczematous patches. Acquired zinc deficiency also typically has ungual involvement (paronychia) and hair involvement (brittle dry strands) and can present secondary to diet changes (alcoholism, severely restricted diet, eating disorders), gastrointestinal disorders (cirrhosis, malabsorption), renal disorders (nephrotic syndrome), drugs, diabetes mellitus, pregnancy, trauma, and hemolytic anemia [14]. Our patient had no cutaneous manifestation in his anogenital or facial regions thus making acquired zinc deficiency a less likely differential.

Primary pellagra is a rare systemic disease caused by niacin (vitamin B3) deficiency. Primary pellagra manifests due to malnutrition often from anorexia nervosa or self-imposed diets. In patients with adequate dietary intake, pellagra can occur via malabsorption in patients with inflammatory bowel disease or post-gastric bypass surgeries. Cutaneously, pellagra presents as dermatitis over sun-exposed skin. Additional systemic symptoms of primary pellagra include diarrhea and dementia [15]. While our patient had the clinical picture of post-gastric bypass and a self-imposed diet, he had no deficiencies of niacin, which makes this diagnosis unlikely.

Progressive symmetrical erythrokeratodermia, or Gottron syndrome, is a rare autosomal-dominant disorder characterized by non-migratory symmetric, erythematous, hyperkeratotic patches on the extremities, buttocks, face, and torso [16]. Progressive symmetrical erythrokeratodermia patients may experience pruritis, but the disorder is otherwise asymptomatic. Histology is non-pathognomonic with acanthosis, orthokeratotic hyperkeratosis, and a prominent granular layer [16]. The presentation of progressive symmetrical erythrokeratodermia is dissimilar to that of our patient, whose eruption was limited to the dorsal hands and lower leg.

The prominent and rapid response to oral zinc therapy that was seen in our patient further supports the diagnosis of NAE. Prior to therapy, our patient had a low-normal zinc level. Early zinc deficiency can present with normal serum zinc levels where deficiencies are present in the epidermis. It is not until the late stages of zinc deficiency that serum zinc levels significantly drop [17]. Interestingly, although mushrooms contain zinc, the patient had a zinc deficiency. This suggests that mushrooms may have a possible “anti-nutrient” profile. However, no literature has been yet published about mushrooms that would support this idea. The proposed mechanism of action of the zinc replacement for treatment of NAE is twofold. First, the oral zinc supplementation corrects the deficiency. Second, zinc supplementation has immunostimulant, antiviral, antioxidant, antiapoptotic, and anti-inflammatory effects [18]. Alternative therapies for NAE, when concurrent with HCV, include treating the underlying HCV with ribavirin and interferon alpha-2 [19].

## Conclusions

Herein, we described a case of a 66-year-old male who was status post gastric bypass surgery and presented with a blistering, scaling, and erythematous rash on dorsal hands and lower legs bilaterally. He was diagnosed with NAE and treated with supplemental zinc therapy. He had resolution of his NAE lesions at follow-up with residual hyperpigmentation. This case underscores the need for proper nutritional education and monitoring in patients following gastric bypass surgery to avoid nutritional deficiencies. Additionally, one should consider including comprehensive nutritional assessment in patient workup to identify deficiencies that may contribute to dermatologic manifestations. Of note, it is important to include this rare condition in the differential diagnosis when patients present with an acral distribution of lichenified violaceous plaques, even when a history of HCV infection is not present.
